# Long Noncoding RNA OR7E156P/miR-143/HIF1A Axis Modulates the Malignant Behaviors of Glioma Cell and Tumor Growth in Mice

**DOI:** 10.3389/fonc.2021.690213

**Published:** 2021-08-06

**Authors:** Haiting Zhao, Peng Du, Renjun Peng, Gang Peng, Jian Yuan, Dingyang Liu, Yi Liu, Xin Mo, Yiwei Liao

**Affiliations:** ^1^Department of Neurosurgery, Xiangya Hospital, The Central South University (CSU), Changsha, China; ^2^Department of Neurology, Xiangya Hospital, The Central South University (CSU), Changsha, China; ^3^Department of Neurosurgery, The Second Affiliated Hospital, Xinjiang Medical University, Urumqi, China

**Keywords:** glioma, hypoxic, lncRNA OR7E156P, miR-143, HIF1A

## Abstract

Gliomas are characterized by high incidence, recurrence and mortality all of which are significant challenges to efficacious clinical treatment. The hypoxic microenvironment in the inner core and intermediate layer of the tumor mass of gliomas is a critical contributor to glioma pathogenesis. In this study, we identified an upregulated lncRNA, OR7E156P, in glioma was identified. The silencing of OR7E156P inhibited cell invasion and DNA synthesis *in vitro* and tumor growth *in vivo*. OR7E156P was intricately linked to the HIF1A pathway. Hypoxia could induce OR7E156P expression, whereas OR7E156P silencing decreased HIF1A protein levels under hypoxic conditions. Hypoxia promoted glioma cell invasion and DNA synthesis, and HUVEC tube formation, whereas OR7E156P silencing partially reversed the cellular effects of hypoxia. HIF1A overexpression promoted, whereas OR7E156P silencing inhibited tumor growth; the inhibitory effects of OR7E156P silencing on tumor growth were partially reversed by HIF1A overexpression. miR-143 directly targeted OR7E156P and HIF1A, respectively. miR-143 inhibition increased HIF1A protein levels, promoted glioma cell invasion and DNA synthesis. Moreover, they enhanced HUVEC tube formation, whereas OR7E156P silencing partially reversed the cellular effects of miR-143 inhibition. HIF1A targeted the promoter region of miR-143 and inhibited miR-143 expression. Altogether a regulatory axis consisting of OR7E156P, miR-143, and HIF1A, was identified which is deregulated in glioma, and the process of the OR7E156P/miR-143/HIF1A axis modulating glioma cell invasion through ZEB1 and HUVEC tube formation through VEGF was demonstrated.

## Introduction

Gliomas are the most common cerebral malignancies (1). Gliomas are characterized by high incidence, recurrence and fatality, with poor prognosis (2–4). Despite advances in treatment regimens such as surgery, radiation therapy and chemotherapy, a satisfactory curative effect has not been achieved as of yet (5). The main research direction is to identify agents inhibiting the invasion and growth of gliomas to ultimately improve the current treatment regimens.

Non-coding RNAs (ncRNAs) can be divided according to their length: short ncRNA, mid-size ncRNA, and long non-coding RNA (lncRNA); they can also be grouped according to their function, loci, and post-transcriptional modification. The oncogenic role of short ncRNAs such as miRNAs have been a long-lasting research topic (6). Contrastingly, the oncogenic role of lncRNAs has only recently been the subject of academic scrutiny (7–9). lncRNA mainly regulates miRNA expression in three ways (10–12): lncRNA competes with target gene mRNA for miRNA binding to counteract the miRNA-mediated suppression on target gene mRNA; lncRNA is spliced to form a miRNA precursor; lncRNA functions as an endogenous miRNA Sponge to inhibit miRNA expression. The mutual regulation between lncRNA and miRNA has been reported in tumors (13–15). Regarding gliomas, it has been revealed by Jia et al. (16) that lncRNA-H19 can regulate glioma angiogenesis and endothelial biological function through the inhibition of miR-29a. Ke et al. (17) reported that lncRNA HOTAIR interacts with miR-326 to regulate glioma cell proliferation and invasion. With the advent of the high-throughput sequencing technique, the identification of deregulated non-coding RNA network in glioma could potentially provide novel targets for glioma therapy.

Hypoxia is one of the most propitious microenvironmental conditions during tumor development, including gliomas (18, 19). In gliomas, glioblastoma multiforme (GBM) is a highly proliferative subtype. The analysis of the phenotypic profile of the regions GBM tumor revealed that the three concentric layers all bear diverse cell phenotypes. Stem cells are localized inside the anoxic and in the intermediate (i.e., hypoxic) area of the tumor mass, whereas more committed cells are scattered among the peripheral and neo-vascularized area (20). The hyperproliferation of cancer cells leads to higher oxygen consumption, exceeding the amount provided by microvessels, thereby disrupting tumor microcirculation and causing hypoxia within the solid tumor (21). The alterations in tumor biology and its microenvironment caused directly or indirectly (via activating transcription factors) by hypoxia could boost the invasive ability of tumors rendering them resistant to chemotherapy and radiotherapy (19, 22). The hypoxic microenvironment is thus regarded as a leading contributor to glioma chemoresistance (23, 24), interfering with angiogenesis, apoptosis, DNA repair, oxidative stress, immune escape, expression and activity of multi-drug resistance related genes. Factors that could potentially be linked with the hypoxic microenvironment should be considered in priority in the selection of non-coding RNA.

In this study, datasets from TCGA and microarray expression profiles from GEO were analyzed for lncRNAs abnormally-expressed in glioma tissues, and lncRNA OR7E156P was selected. The specific effects of OR7E156P on glioma cell invasion, DNA synthesis, and HUVEC tube formation were examined. The signaling pathways potentially associated with OR7E156P were analyzed and HIF1A signaling was identified. Then, the *in vitro* effects of OR7E156P upon the invasion and proliferation, and HUVEC (human umbilical vein endothelial cell) tube formation ability were examined under normoxia or hypoxia, and the dynamic effects of OR7E156P and HIF1A on tumor growth in nude mice *in vivo* were examined. Next, miRNAs which could simultaneously bind to OR7E156P and HIF1A were selected, and the putative bindings were subsequently examined. The dynamic effects of OR7E156P and miR-143 on HIF1A levels, glioma cell invasion and proliferation, and HUVEC tube formation were examined under conditions of normoxia or hypoxia. The binding of HIF1A to miR-143 promoter region and HIF1A regulation of miR-143 were ultimately examined. A network formed by lncRNA OR7E156P, miR-143, and HIF1A that might modulate hypoxia-related glioma growth and metastasis was conclusively demonstrated.

## Materials and Methods

### Clinical Sampling

A total of ten glioma tissues from the tumor core region (core), ten glioma tissues from the tumor intermediate region (intermediate), and ten tissue samples from the peritumoral brain edema (PTBE) region were harvested from patients who underwent surgical resection at Xiangya Hospital. The sampling procedure was conducted with the approval of the Ethics Committee of Xiangya Hospital. All the tissue samples were stored at -80°C pending further laboratory investigations.

### Cell Lines and Cell Culture

The human fetal glial cell line SVG p12 (ATCC^®^ CRL-8621^™^), as a normal control, was procured from ATCC (Manassas, VA, USA) and cultured in Eagle’s Minimum Essential Medium (Catalog No. 30-2003, ATCC). Human glioma cell line T98G (CRL-1690™) was obtained from ATCC and cultured in Eagle’s Minimum Essential Medium (ATCC) and supplemented with 10% fetal bovine serum (FBS; Invitrogen, Waltham, MA, USA). Human glioblastoma cell line U87-MG was procured from the Shanghai Institutes for Biological Sciences Cell Resource Center (Shanghai, China) and cultured in high-glucose Dulbecco’s modified Eagle medium (DMEM, GIBCO, Carlsbad, CA, USA) supplemented with 10% FBS. Human glioblastoma cell line LN229 was obtained from the Institute of Biochemistry and Cell Biology, Chinese Academy of Science (Shanghai, China) and cultured in DMEM/F12 1:1 medium (Corning, Corning, NY, USA) supplemented with 10% FBS. Human glioma cell line U251-MG was obtained from Sigma-Aldrich (09063001; St. Louis, MO, USA) and cultured in DMEM (GIBCO) supplemented with 10% FBS. Primary umbilical vein endothelial cells (HUVEC; PCS-100-010^™^) were obtained from ATCC (Manassas, VA, USA). All the cells were cultured in 5% CO_2_ saturation at a temperature of 37°C.

The primary human glioma cell-line was established and cultured from high-grade glioma patients’ tumor (25). Fresh human glioma tissues were thoroughly washed (in cold PBS), minced and filtered. Single-cell suspensions were obtained through digestion, and primary glioma cells were cultured in in DMEM medium (GIBCO), supplemented with a 10% FBS, penicillin/streptomycin solution (1:100, Sigma-Aldrich) and 4 mML-glutamine (Sigma-Aldrich). The phenotypes of cell lines were photographed using microphotography (26). Moreover, the cell morphology of glioma cell was evaluated with the label of GFAP and S100B proteins, and the GFAP and S100B protein expressions were detected by Immunocytochemistry (27).

### Cell Transfection

The knockdown of lncRNA OR7E156P was conducted through the transduction of lentivirus containing short hairpin RNA (shRNA) specific for lncRNA OR7E156P (Lsh1/2-OR7E156P; Ori-Bio, Changsha, China). The sequences of OR7E156P shRNA listed in **Table 1**.

**Table 1:** The sequence of shRNA OR7E156P and miR-143-5p mimics and inhibitor.

Gene	Sequence (5’-3’)
shRNA-NC	Top Strand: GATCCACACAGCAGGTCAAGAGGAGTC
	TCGAGACTCCTCTTGACCTGCTGTGTTTTTTG
	Bottom Strand: AATTCAAAAAACACAGCAGGTCAAG
	AGGAGTCTCGAGACTCCTCTTGACCTGCTGTGTG
sh-OR7E156P#1	Top Strand: GATCCGGCTATAGTAGATTCCAAATA CTCGAG TATTTGGAATCTACTATAGCCTTTTTG
	Bottom Strand: AATTCAAAAAGCTATAGTAGATTC CAAATA CTCGAG TATTTGGAATCTACTATAGCCG
sh-OR7E156P#2	Top Strand: GATCC CTGTCTAGTTGTTCAGAGACG CTCGAG CGTCTCTGAACAACTAGACAG TTTTTG
	Bottom Strand: AATTCAAAAACTGTCTAGTTGTTCA GAGACGTCGAGCGTCTCTGAACAACTAGACAGG
mimics NC	UUCUCCGAACGUGUCACGUTT
miR-143-5p mimics	GGUGCAGUGCUGCAUCUCUGGU
inhibitor NC	CAGUACUUUUGUGUAGUACAA
miR-143-5p inhibitor	ACCAGAGAUGCAGCACUGCACC

HIF-1α-overexpressing plasmid (HIF-1α-cDNA) was constructed through the insertion of the full-length HIF-1α cDNA into the pcDNA3.1 expression vector. The overexpressing plasmid was verified by DNA sequencing.

### PCR-Based Analysis

The total RNA was extracted from target cells or tissues using a Trizol reagent (Invitrogen) as stipulated by the manufacturer’s instructions and then was reverse transcribed using the First-strand cDNA synthesis kit (Promega). The expression levels were then examined using the SYBR Green PCR Master Mix (Qiagen) as per the manufacturer’s instructions. Normalization was performed using Tubulin (for lncRNAs and mRNAs) or U6 (for miRNAs). Relative expression values were calculated using the 2^-ΔΔCt^ method. The specific primer sequences used are listed in **Table 2**.

**Table 2:** The primer sequence for PCR assay.

Gene	Forward	Reverse
CYB561D2	CTGGAACAGGAGGGGAGGGGTG	CGGGCAGGTGTGGAAATAAGCA
DLEU1	GTTGGAGGTAAACAAATACGGGTC	AAGGTCTCATTGATACATTCCGTTG
HCP5	GGGGTCTGAAGGATGGTGACTGCG	ACACTGCCTGGTGAGCCTGTTTGA
OR7E156P	CAACTTGATTGCCTTACAAATGACC	GGGAGTTGAGAAGGGTCACAGA
HIF1A	GAACGTCGAAAAGAAAAGTCTCG	CCTTATCAAGATGCGAACTCACA
VEGF	AGGGCAGAATCATCACGAAGT	AGGGTCTCGATTGGATGGCA
ZEB1	ACAACCAAGTGCAGAAGA	CATTTGCAGATTGAGGCTGA
Snail	TCGGAAGCCTAACTACAGCGA	AGATGAGCATTGGCAGCGAG
Vimentin	AGTCCACTGAGTACCGGAGAC	CATTTCACGCATCTGGCGTTC
miR-17	GCACTGCAGTGAAGGCAC	CAGTGCGTGTCGTGGA
miR-20a	GCCGTAAAGTGCTTATAGTGC	CAGTGCGTGTCGTGGA
miR-153	GCCTTGCATAGTCACAAAA	CAGTGCGTGTCGTGGA
miR-519d	GCAAAGTGCCTCCCTTT	CAGTGCGTGTCGTGGA
miR-143-5p	GGTGCAGTGCTGCATC	CAGTGCGTGTCGTGGA
Tubulin	TGGACTCTGTTCGCTCAGGT	TGCCTCCTTCCGTACCACAT
U6	CTCGCTTCGGCAGCACA	AACGCTTCACGAATTTGCGT

### Histological Analyses

Hematoxylin and eosin (H&E) and immunohistochemical (IHC) staining were performed as previously described (28). The glioma tissues samples were put in 4% paraformaldehyde solution overnight, and were subsequently dehydrated in ethanol gradient, embedded in paraffin, and sliced into cross-sections of 5 μm. For H&E staining, the section samples were stained using hematoxylin and eosin after deparaffinage. The slices were then mounted and observed under a light microscope (Leica Microsystems, Wetzlar, Germany). For IHC staining, the sections were briefly incubated with 0.3% H_2_O_2_ for 15min and further incubated overnight with mouse polyclonal primary antibody of HIF-1α (ab1; Abcam, Cambridge, CA, USA). The sections were subsequently incubated with 45µl secondary antibody horseradish peroxidase-conjugated goat polyclonal anti-mouse IgG H&L (HRP) (ab6789, Abcam) at 37°C for 30 min. Slices were stained with 3, 3’-diaminobenzidine (DAB) working solution for 3 min, then washed in water for 10 min. The slices were counterstained with hematoxylin. After rewashing the slices in water for 10 minutes, the sections were finally dehydrated and mounted. The slices were ultimately observed under a microscope (Leica Microsystems).

### ELISA

The amount of VEGF was determined using a Human VEGF Quantikine ELISA Kit (DVE00; R&D System, Minneapolis, MN, USA) in a DuoSet^®^ ELISA Development Systems (R&D System) as per manufacturer’s instructions and aforementioned methods (29).

### Immunoblotting

The protein levels of HIF-1α, VEGF, ZEB1, Snail, and Vimentin in target cells were detected by Immunoblotting. Target cells were resuspended in an equal volume of a RIPA lysis buffer (30). The suspension was placed on ice for 15 min, vortexed for 10 s and centrifuged for 10 min at 12,000 rpm at 4°C. The supernatant was removed and protein samples were then collected. The concentration of the protein samples was assessed using the Bio-Rad Protein Assay kit (Bio-Rad, Hercules, CA, USA). The protein samples were then loaded onto a SDS-PAGE minigel for protein separation and then transferred onto PVDF membranes. The proteins were then probed with the following antibodies: anti-HIF-1α (ab1, Abcam), VEGF (ab1316, Abcam), ZEB1 (ab203829, Abcam), Snail (ab53519, Abcam), and Vimentin (ab92547, Abcam) at 4°C overnight, and incubated with HRP-conjugated secondary antibody (1:5000). Signals were visualized using ECL Substrates (Millipore, USA). The protein expression was normalized to endogenous Tubulin.

### Luciferase Activity

The predicted miR-143-5p binding to OR7E156P or 3’UTR of HIF1A was verified using a Luciferase reporter assay. The wild-type or mutated OR7E156P or 3’UTR of HIF1A was cloned to the downstream of the Renilla psiCHECK2 vector (Promega, Madison, WI, USA), named wt-OR7E156P or wt-HIF1A 3’UTR or mut-OR7E156P or mut-HIF1A 3’UTR. 293T cells were subsequently co-transfected with two types of luciferase reporter vectors and miR-143-5p mimics/miR-143-5p inhibitor and the luciferase activity was examined using the Dual-Luciferase Reporter Assay System (Promega). The sequences of miR-143-5p mimics and miR-143-5p inhibitor listed in **Table 1**.

For HIF-1α modulating miR-143 transcriptional activity, wildtype (psicheck-2-pro-miR-143) and mutant reporter constructs (psicheck-2-pro-miR-143-mut) were structured following the aforementioned methods (31). A miR-143 promoter fragment containing the hypoxia response element (HRE) was cloned into pGL3 luciferase vector to generate the wildtype reporter construct. Mutation at any binding sites in the HRE motifs was introduced into pGL3 luciferase vector with the QuikChange Site-Directed Mutagenesis Kit (Stratagene, La Jalla, CA, USA). Both of the constructs were verified by sequencing. Cells were subsequently co-transfected with HIF-1α-cDNA and psicheck-2-pro-miR-143 or psicheck-2-pro-miR-143-mut and pRL-cmv (Renilla luciferase). The transactivation potential was tested through the Dual-Glo Luciferase Assay System (Promega, Madison, WI, USA) by measuring luciferase activity after 48h.

### RNA Immunoprecipitation (RIP)

293T cell lysates were used for RIP. The Imprint RNA Immunoprecipitation Kit (Sigma, USA) was used in RNA immunoprecipitation with the AGO2 antibody (ab32381, Abcam, USA), which is a key component of the microRNA-containing RISC complex. AGO2 were used as positive controls and IgG as the negative controls. The levels of miR-143, OR7E156P, and HIF1A in the precipitates were determined using a real-time PCR.

### Cell Invasive Capacity Evaluated by Transwell Assay

Target cells were planted in medium without serum on the top side of polycarbonate Transwell filters coated with Matrigel at a density of 5 × 10^5^. The medium with serum was fixed in the bottom chamber. The cells were incubated at a temperature of 37°C for 48 h. Non-invaded cells in the top chambers were removed with cotton swabs. Invaded cells on the lower membrane surface were fixed in 100% methanol for 10 min, air-dried, stained with crystal violet solution, and then quantified under a microscope.

### DNA Synthesis Ability Examined by EdU Assay

DNA synthesis is based on the method of incorporating thymidine analogue 5-ethoxy 2-deoxyuridine (EdU) into genomic DNA using the Click-IT EdU Alexa Fluor 488 kit following the aforementioned method (32, 33). Apollo staining and DAPI staining were performed and EdU positive cells were observed under a fluorescence microscope. The incorporation rate of EdU was calculated and quantified as the ratio of EdU-positive cells (green cells) to total DAPI-positive cells (blue cells) through ImageJ software (Media Cybernetics, Silver Springs, MD, USA).

### HUVEC Tubule Formation Ability

Glioma cells were infected with Lsh-NC or Lsh-OR7E156P, exposed to normoxia, and the culture medium were collected and used as a conditional culture medium (U251-MG CM and U87-MG-CM). HUVECs were then seeded into 96-well microtiter plates coated with Matrigel (80 μl/well), incubated for 1 h, and then suspended in above-mentioned conditional culture medium, respectively. The formation of tube-like structures was examined 4-6 h later. Tube formation was analyzed by quantifying the number of branches or tube length per high-powered field using a ImageJ software (NIH, Bethesda, MD, USA).

### Tumorigenicity Assay

4 weeks old Babl/c nude mice were purchased from the SLAC experimental animal center (Changsha, China). All animal procedures stringently followed the ethical guidelines imposed by the Xiangya Hospital. For tumor establishment, glioma cells were infected or co-infected with Adv-HIF1A (Adv-NC as control) and/or Lsh-OR7E156P (Lsh-NC as control) and then the mice were injected subcutaneously with 2.5× 10^5^ cells in a 200-μl total volume into their right dorsolateral flanks. After 5 weeks, the mice were sacrificed and the tumor volume was calculated as per the following formula: tumor volume (mm^3^) = length × width^2^/2. The tumor tissues were subsequently formalin-fixed and paraffin-embedded alongside standard procedures for histological observation.

### Statistics Analysis

The results from at least three independent experiments were processed with GraphPad (San Diego, CA, USA) and expressed as mean ± standard deviation (S.D.). Statistical significance was evaluated by a one-way analysis of variance (ANOVA) followed by a Tukey’s multiple comparison test or by a Student’s *t*-test. A *P* value of less than 0.05 was deemed statistically significant.

## Results

### Selection of lncRNAs Related to Glioma Carcinogenesis

**Figure 1:**
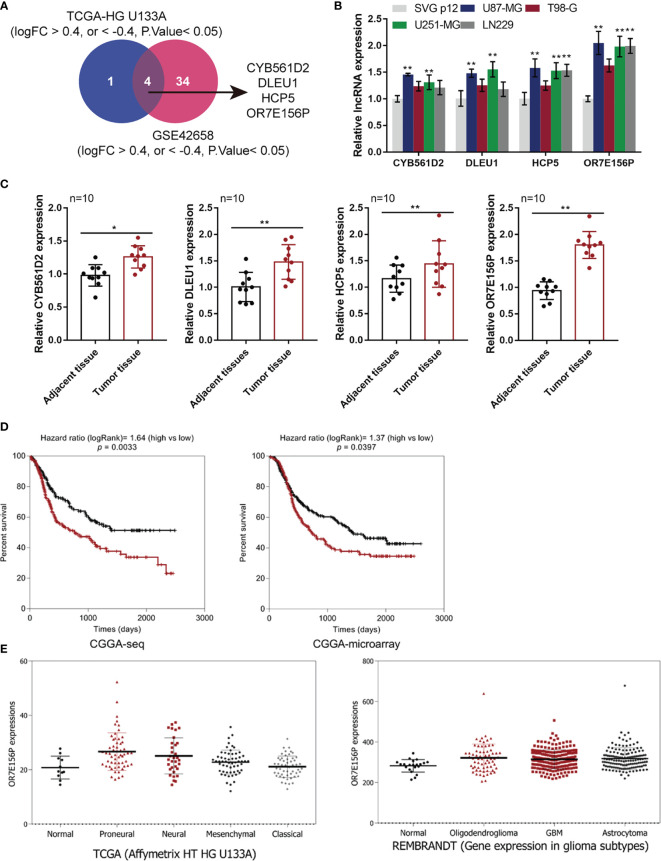
Selection of lncRNAs related to glioma carcinogenesis **(A)** TCGA-GBM microarray dataset, AffyU133a, from Xena (http://xena.ucsc.edu/) and GSE42658 were used to select lncRNAs related glioma carcinogenesis. TCGA-HG U133UA reported differentially-expressed genes between U251-MGU251-MGglioma tissues and normal non-cancerous tissues (|logFC| > 0.4, *P* < 0.05); GSE53014 reported differentially-expressed genes in 5 cases glioma tissues and 8 cases normal non-cancerous tissues (|logFC| > 0.4, *P* < 0.05). After cross-check, CYB561D2, DLEU1, HCP5, and OR7E156P exhibited similar expression trend. **(B)** The expression of CYB561D2, DLEU1, HCP5, and OR7E156P was determined in SVG p12, U87-MG, T98-G, U251-MG, and LN229 cells by qRT-PCR. **(C)** The expression of CYB561D2, DLEU1, HCP5, and OR7E156P was determined in 10 paired tumor and adjacent non-cancerous tissue samples by qRT-PCR. **(D)** Cases from the Chinese Glioma Genome Atlas (CGGA)-seq or CGGA-microarray were divided into two groups, respectively, using the median value of OR7E156P expression as cut-off. The correlation between OR7E156P expression and the survival in patients with gliomas was analyzed using a Cox proportional hazard regression model and log-rank analysis and the results were shown as Kaplan-Meier survival curves. **(E)** OR7E156P expression in different tissues or different glioma subtypes based on the RNA-seq data from the Human Cancer Atlas database (The Cancer Genome Atlas, TCGA) and the REMBRANDT database (http://betastasis.com/glioma/rembrandt/). *p < 0.05, **p < 0.01.

Two online datasets were selected and analyzed, the TCGA-GBM microarray dataset (AffyU133a) from Xena (http://xena.ucsc.edu/) and the GSE42658, to identify lncRNAs related to glioma carcinogenesis using bioinformatics analysis. A differential expression analysis was performed in 10 cases of para-cancer control tissues and 197 cases of GBM tumor tissues from TCGA-GBM microarray dataset (AffyU133a) using an R language toolkit limma, and 8 differentially expressed ncRNAs were yielded (|logFC| > 0.4, *P* < 0.05), among which 3 were down-regulated (CDR1, MEG3, PART1) and 5, up-regulated (CYB561D2, DLEU1, DLEU2, HCP5, OR7E156P) (**Figure S1**). A total of 5 glioma tissues and 8 non-cancerous tissues were selected from GSE42658 U251-MGU251-MG and 61 ncRNAs were found to be significantly differentially-expressed (|logFC| > 0.4, *P* < 0.05), among which 24 were down-regulated and 37 up-regulated (**Figure S2**). After cross-checking, CYB561D2, DLEU1, HCP5, and OR7E156P were selected due to their similar expression trend in both datasets (**Figure 1A**). For further confirmation, the expression of CYB561D2, DLEU1, HCP5 and OR7E156P were examined in a human fetal glial cell line, SVG p12, and four glioma cell lines, U87-MG, T98-G, U251-MGU251-MG MG, and LN229; **Figure 1B** reveals that the expression of all the four lncRNAs was significantly upregulated in all the four glioma cell lines compared with that in the SVG p12 cell line; among the four lncRNAs, OR7E156P was more upregulated. In tissue samples (clinical characteristics listed in **Table 3**), the expression of all the four lncRNAs was significantly upregulated in glioma tissues compared to that in adjacent non-cancerous tissues, OR7E156P more upregulated (**Figure 1C**). As a further confirmation, a survival analysis using a Cox proportional hazard regression model and log-rank analysis were performed. According to the RNA-seq data or microarray expression profile data from Chinese Glioma Genome Atlas (CGGA), high OR7E156P expression was correlated with a lower survival percentage (**Figure 1D**). Moreover, according to the RNA-seq data from the Human Cancer Atlas database (The Cancer Genome Atlas, TCGA) and the REMBRANDT database (http://betastasis.com/glioma/rembrandt/), OR7E156P expression was higher in glioma subtypes than that in non-cancerous tissue samples (**Figure 1E**). Therefore, lncRNA OR7E156P could be potentially associated with glioma pathogenesis, and was selected for further experimentation.

**Table 3:** Clinical characteristics of patients enrolled for sampling.

Number	age	gender	Peritumoral brain edema	Tumor size (cm)	WHO grading	Tumor location	KPS	Survival state
1	65	male	obvious	3.6×3.0	4	The left side of the thalamus	40	waking state
2	55	male	obvious	8.5×4.5	2	The left temporal occipital lobe	70	waking state
3	30	male	obvious	4.4×4.5	4	The left frontal lobe	50	waking state
4	66	male	obvious	7.3×4.3	4	The left parietal occipital lobe	70	waking state
5	54	female	obvious	7.2×4.5	4	The left temporal lobe	100	waking state
6	63	male	obvious	5.5×4.5	4	The left frontal lobe	30	comatose state
7	10	female	little	7.5×4.3	2	epencephalon	70	waking state
8	54	female	obvious	2.7×6.5	4	The right temporal parietal lobe	70	waking state
9	33	female	little	5.3×3.9	3	The right insular lobe	80	waking state
10	28	female	little	3.0×3.8	2	The right basal ganglia region	60	waking state

### *In Vitro* and *In Vivo* Effects of OR7E156P on Glioma Cells

**Figure 2:**
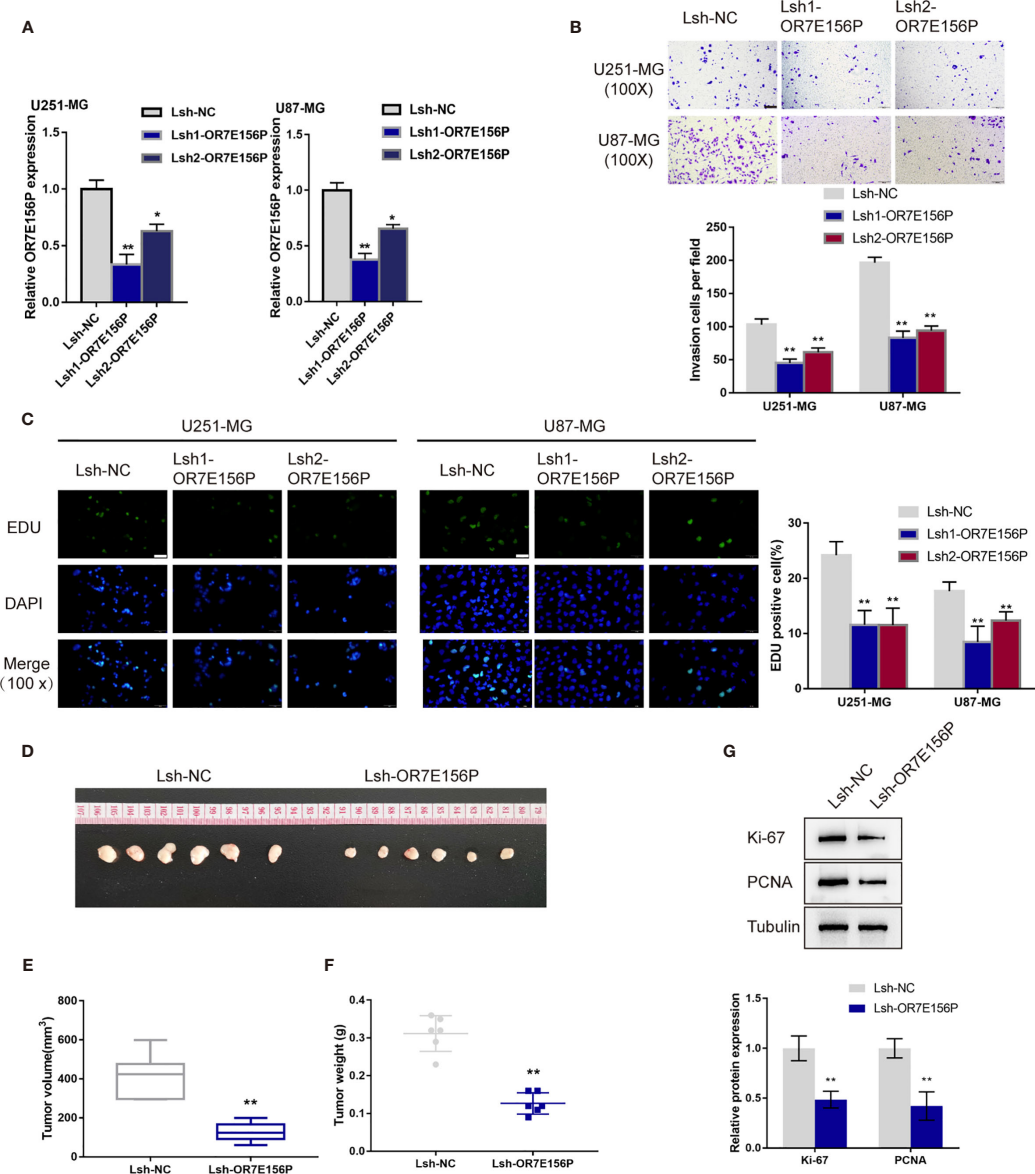
*In vitro* and *in vivo* effects of OR7E156P on glioma **(A)** OR7E156P silencing was achieved in U251-MG and U87-MG cells by infecting cells with lentivirus containing short hairpin RNA for OR7E156P (Lsh1-OR7E156P or Lsh2-OR7E156P). Lsh-NC was infected as a negative control. The silencing efficiency was verified by qRT-PCR. Then, U251-MG and U87-MG cells were infected with Lsh1-OR7E156P, Lsh2-OR7E156P, or Lsh-NC and examined for cell invasion by Transwell assay **(B)**; DNA synthesis capacity by EdU assay **(C)**. **(D)** Subcutaneously implanted tumor model was constructed in nude mice by injecting glioma cells infected with Lsh1-OR7E156P or Lsh-NC. **(E, F)** Tumor volume and tumor weight were examined in each group. **(G)** The protein levels of Ki-67 and PCNA in tumor tissues were examined by Immunoblotting. *P < 0.05, **P < 0.01.

To confirm the bioinformatics analysis, OR7E156P silencing was generated in U251-MGU251-MG MG and U87-MG cells by infecting them with lentivirus containing short hairpin RNA for OR7E156P (Lsh1-OR7E156P or Lsh2-OR7E156P). Lsh-NC was infected as a negative control. The silencing efficiency was verified by a qRT-PCR and it was established that Lsh1-OR7E156P exhibited better efficiency (**Figure 2A**). U251-MGU251-MG MG and U87-MG cells were subsequently infected with Lsh1-OR7E156P, Lsh2-OR7E156P, or Lsh-NC, and examined for related indexes. As illustrated in **Figures 2B, C**, OR7E156P silencing significantly inhibited the cell invasion and DNA synthesis capacity of both glioma cell lines.

Firstly, the primary human glioma cell was established and cultured from high-grade glioma patients’ tumor. As presented in **Figure S3A**, the morphology of the primary tumor cells is depicted. Moreover, the cell morphology of primary human glioma cells, U251-MG and U87-MG cells were evaluated through the label of GFAP and S100B proteins (**Figure S3B**). These results suggested the successful establishment of the primary human glioma cell. The effects of OR7E156P on the primary human glioma cells’ proliferation and invasion were also subsequently investigated. The primary human glioma cells were infected with Lsh1-OR7E156P, Lsh2-OR7E156P, or Lsh-NC and the transfection efficiency was detected by a qRT-PCR assay and Lsh1-OR7E156P exhibited better efficiency (**Figure S3C**). As illustrated in **Figures S3D, E**, OR7E156P silencing significantly inhibited the cell invasion and DNA synthesis capacity of primary human glioma cell.

Regarding the *in vivo* effects, subcutaneously implanted tumor model was constructed in nude mice by injecting glioma cells infected with Lsh1-OR7E156P or Lsh-NC (**Figure 2D**). Consistent with the *in vitro* findings, OR7E156P silencing reduced tumor volume and tumor weight (**Figures 2E, F**). Meanwhile, the protein levels of Ki-67 and PCNA in tumor tissues derived from OR7E156P silenced glioma cells were significantly decreased compared to those in tumors derived from Lsh-NC-infected glioma cells (**Figure 2G**).

### Expression and Association of OR7E156P and HIF1A Under Normoxia or Hypoxia Conditions

**Figure 3:**
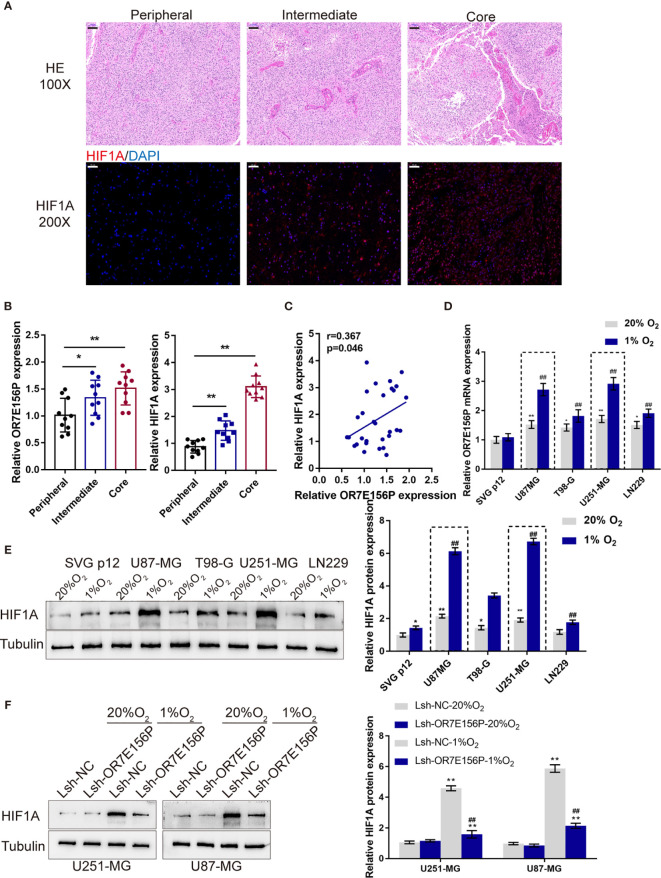
Expression and association of OR7E156P and HIF1A under normoxia or hypoxia conditions **(A)** Glioma tissues samples from the core region, intermediate region, and the peripheral region were collected and examined by Hematoxylin and eosin (H&E) staining for histopathological characteristics and the immunofluorescence (IF) staining for the protein content and distribution of HIF1A. **(B)** The expression of HIF1A and OR7E156P in glioma tissues samples from the core region, intermediate region, and the peripheral region was examined using real-time PCR. **(C)** The correlation of HIF1A and OR7E156P expression in tissue samples was analyzed using Pearson’s correlation analysis. **(D, E)** A human fetal glial cell line SVG p12 and four glioma cell lines, U87-MG, T98-G, U251-MG, and LN229 were exposed to 1% or 20% oxygen and examined for the expression of OR7E156P using real-time PCR and protein levels of HIF1A using Immunoblotting. **(F)** U251-MG and U87-MG cells were transfected with Lsh-OR7E156P, exposed to 1% or 20% oxygen, and then examined for the protein levels of HIF1A by Immunoblotting. *P < 0.05, **P < 0.01, ^##^ *P* < 0.01.

As mentioned above, hypoxia and hypoxia-induced expression of HIF1A play a critical role in glioma development and aggressiveness (23, 24). To investigate the mechanism of OR7E156P functions, the BioCarta tool from (https://www.gsea-msigdb.org/gsea/) was used to analyze the association between OR7E156P and HIF signaling pathway; as shown in **Figure S4**, BIOCARTA_HIF_PATHWAY was significantly correlated with OR7E156P. Consequently, the levels of OR7E156P and HIF1A were then examined in tissue samples and glioma cell lines in conditions of normoxia and hypoxia. The histopathological features of the core region, intermediate region, and peripheral region were examined through H&E staining. These three regions showed to be defined by distance from the hypoxic central core in the determination of the molecular and phenotypic characteristics associated with the hypoxic concentric gradient. The intermediate portion is a thin transition area between the partially necrotic core and the peripheral area, defined by the presence of tumor angiogenesis (**Figure 3A**). The expression of HIF1A and OR7E156P was higher in the intermediate region and highest in the core region compared to that in the peripheral region (**Figure 3B**). Within tissues, HIF1A expression exhibited a positive correlation with OR7E156P expression (**Figure 3C**). Secondly, a human fetal glial cell line SVG p12 and four glioma cell lines, U87-MG, T98-G, U251-MG, and LN229, were exposed to 1% or 20% oxygen saturations, HIF1A protein levels and OR7E156P expression levels were subsequently determined. As illustrated in **Figures 3D, E**, hypoxia (1% O_2_) significantly induced HIF1A protein levels, especially in U251-MG and U87-MG cells; the expression of OR7E156P showed to be dramatically upregulated within glioma cells than that within HEB cells, more upregulated in U251-MG and U87-MG cells. Next, U251-MG and U87-MG cells were infected with Lsh-OR7E156P, exposed to 1% or 20% oxygen saturations, and the protein levels of HIF1A were then examined. Hypoxia significantly induced the protein levels of HIF1A in both glioma cell lines compared to those in cells exposed to conditions of normoxia (#*P*<0.05, ##*P*<0.01), while OR7E156P knockdown significantly decreased hypoxia-induced increase in HIF1A protein (*P<0.05, **P<0.01; **Figure 3F**). Therefore, HIF1A could potentially be involved in the functions of OR7E156P on glioma cell aggressiveness. Moreover, U251-MG and U87-MG cell lines were selected for further experiments.

### Dynamic Effects of HIF1A and OR7E156P on Glioma *In Vitro* and *In Vivo*

**Figure 4:**
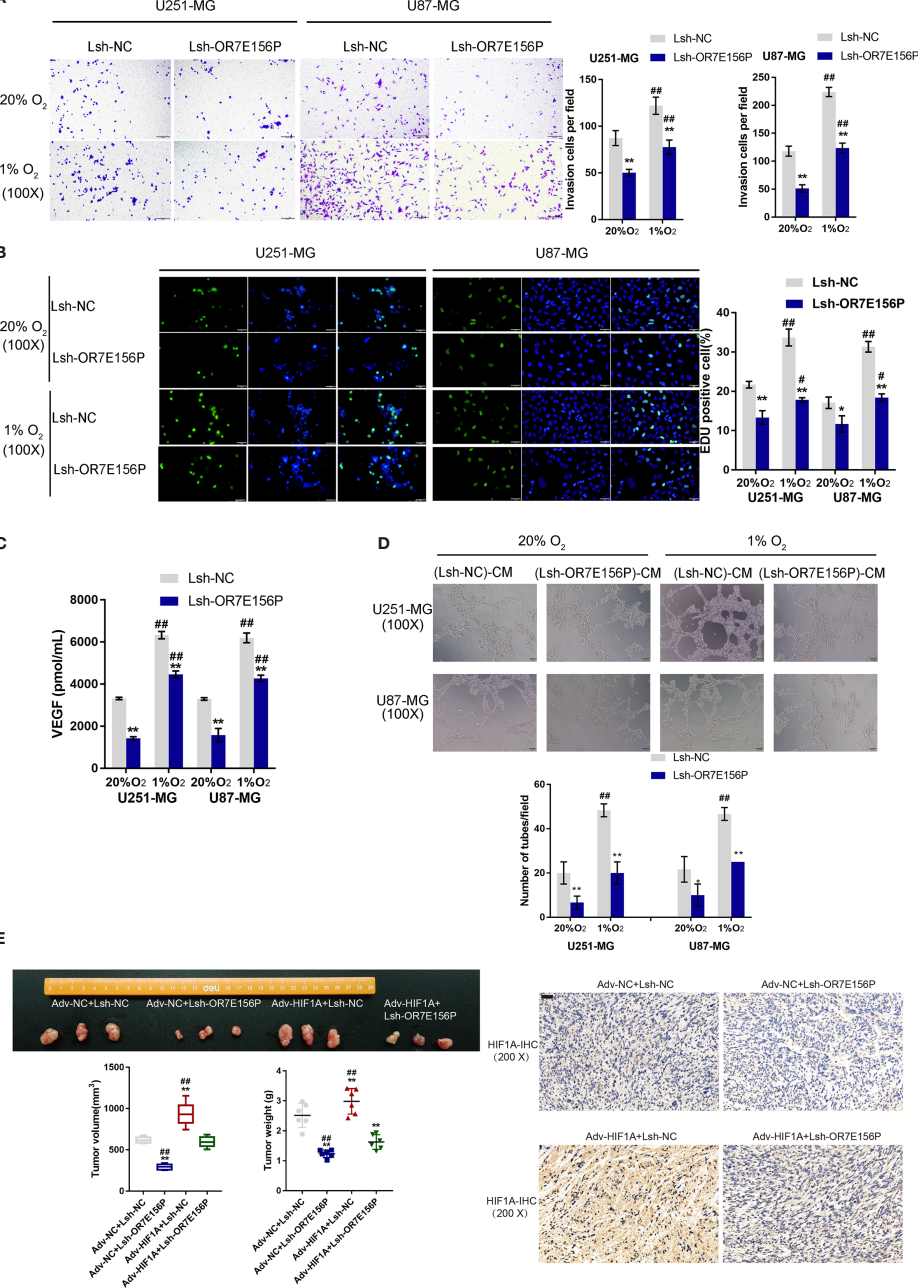
Dynamic effects of HIF1A and OR7E156P on glioma *in vitro* and *in vivo* U251-MG and U87-MG cells were transduced with Lsh-OR7E156P or Lsh-NC, exposed to 1% O_2_ or 20% O_2_, and examined for cell invasion by Transwell **(A)** and DNA synthesis capacity by EdU **(B)**. **(C)** Conditioned medium was obtained from Lsh-OR7E156P- or Lsh-NC-infected U251-MG and U87-MG cells and used for human umbilical vein endothelial cell (HUVEC) culturing; the content of VEGF in culture medium was determined by ELISA. **(D)** The tube formation capacity of HUVEC in different conditional culture medium was determined. **(E)** Tumor volumes and weights from mice bearing tumors derived from glioma cells infected or co-infected with Adv-HIF1A (Adv-NC as control) and/or Lsh-OR7E156P (Lsh-NC as control) were measured and calculated. HIF-1α protein content and distribution in tumor tissues was examined by IHC staining. *P < 0.05, **P < 0.01, ^#^p < 0.05, ^##^ *P* < 0.01.

To investigate whether OR7E156P participates in the functions of HIF1A on glioma cell aggressiveness, next, U251-MG and U87-MG cells were transduced Lsh-OR7E156P or Lsh-NC, exposed to 1% O_2_ or 20% O_2_, and examined cell invasion and DNA synthesis capacity. As illustrated in **Figures 4A, B**, hypoxia significantly promoted cancer cell invasion and DNA synthesis, whereas OR7E156P silencing significantly attenuated the promotive effects of hypoxia on cancer cells. Next, a conditioned medium was obtained from Lsh-OR7E156P- or Lsh-NC-infected U251-MG and U87-MG cells and used for HUVEC culturing; the tube formation capacity of HUVEC in different conditional culture media was subsequently determined. Hypoxia significantly increased, whereas OR7E156P silencing partially decreased the content of VEGF in culture medium (**Figure 4C**). Similarly, the hypoxic condition (1% O_2_ saturation) significantly promoted the tube formation by HUVECs, whereas OR7E156P silencing partially inhibited HUVEC tube formation (**Figure 4D**).

Regarding the dynamic effects of HIF1A and OR7E156P on tumor growth, a subcutaneously implanted tumor model was constructed in nude mice by injecting glioma cells transduced with Adv-HIF1A (Adv-NC as control) and/or Lsh1-OR7E156P (Lsh-NC as control). As illustrated in **Figure 4E**, the tumor volumes and weights in mice bearing tumor derived from Adv-NC + Lsh1-OR7E156P co-infected cells were the lowest; contrariwise, those in mice bearing tumor derived from Adv-HIF1A + Lsh-NC co-infected cells were the highest (**Figure 4E**). OR7E156P knockdown partially reversed the promotive effects of HIF1A overexpression on tumor growth (**Figure 4E**). Consistently, IHC staining revealed that HIF-1α protein content was the highest in Adv-HIF1A + Lsh-NC tumors while it was decreased in Adv-NC + Lsh1-OR7E156P tumors (**Figure 4E**). These data suggest that HIF1A overexpression could partially reverse the inhibitory effects of OR7E156P knockdown on tumor growth.

### miR-143 Contributes to the Interaction of OR7E156P to HIF1A

**Figure 5:**
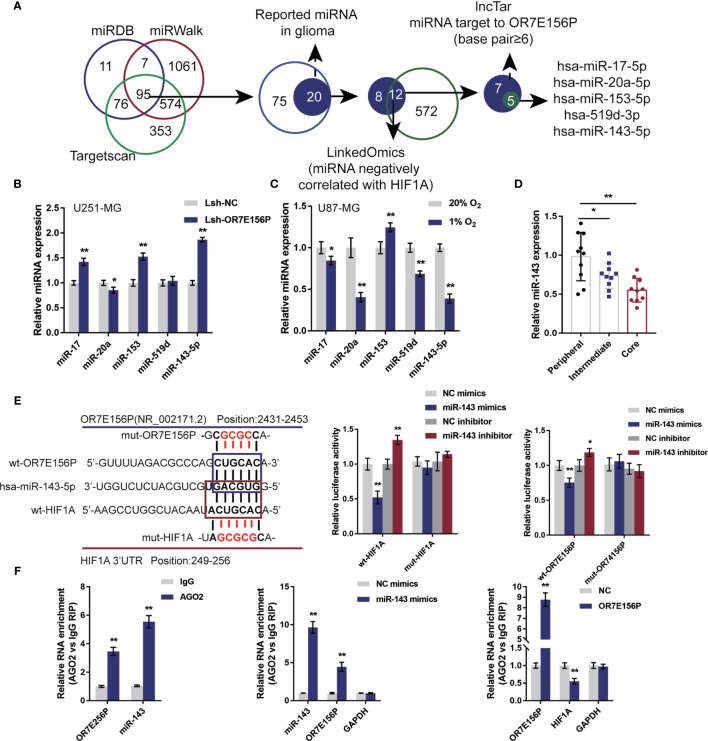
miR-143 is involved in the cross-talk between OR7E156P and HIF1A **(A)** A schematic diagram showing the selection of miRNAs related to glioma and might simultaneously target OR7E156P and HIF1A. Five miRNAs were selected: miR-17, miR-20a, miR-153, miR-519d, and miR-143-5p. **(B)** U251-MG and U87-MG cells were infected with Lsh-OR7E156P and examined for the expression of miR-17, miR-20a, miR-153, miR-519d, and miR-143-5p by real-time PCR. **(C)** U251-MG and U87-MG cells were exposed to 1% or 20% oxygen and examined for the expression of miR-17, miR-20a, miR-153, miR-519d, and miR-143-5p by real-time PCR. **(E)** Wild-type and mutant-type OR7E156P or HIF1A 3’UTR luciferase reporter vectors were constructed as described in the *Materials and Methods* section. These vectors were co-transfected in 293T cells with miR-143 mimics or miR-143 inhibitor. The luciferase activity was determined. **(F)** RIP assays were performed to confirm the binding of miR-143 to OR7E156P and the 3’-UTR of HIF1A using AGO2 antibody. The levels of miR-143, OR7E156P, and HIF1A in precipitated AGO2 proteins were examined using real-time PCR. *P < 0.05, **P < 0.01.

Mechanistically, it is proposed that lncRNAs could act as miRNA “sponges”, inhibiting normal miRNA targeting activity on mRNA (34, 35). Next, the study continued to screen for miRNAs that could potentially be related to both OR7E156P and HIF1A, thus mediating the crosstalk between OR7E156P and HIF1A. As illustrated in **Figure 5A**, miRDB, miRwalk, and Targetscan were used to yield miRNAs targeting HIF1A and all the three tools predicted that 95 miRNAs could bind to HIF1A. Among them, twelve of the 20 miRNAs were found to be negatively correlated with HIF1A in gliomas on the linkedOmics website. Next, among these 12 miRNAs, 5 miRNAs were predicted to obtain the binding site to OR7E156P by the online tool lncTar (**Figure 5A**). These 5 miRNAs (miR-17, miR-20a, miR-153, miR-519d, and miR-143-5p) could potentially possess binding sites with OR7E156P/HIF1A, and are negatively correlated with HIF1A in glioma, respectively.

To further select the candidate miRNAs, U251-MG cells were infected with Lsh-OR7E156P and examined for miR-17, miR-20a, miR-153, miR-519d, and miR-143-5p expression; as illustrated in **Figure 5B**, miR-17, miR-153, and miR-143-5p were significantly upregulated by OR7E156P knockdown. Moreover, U87-MG cells were exposed to 1% or 20% oxygen and miR-17, miR-20a, miR-153, miR-519d, and miR-143-5p expression were determined; as shown in **Figure 5C**, miR-17, miR-20a, miR-519d, and miR-143-5p were significantly downregulated under hypoxia, miR-20a and miR-143-5p more downregulated. More importantly, miR-143-5p expression was significantly downregulated in glioma core and intermediate regions compared to the peripheral region, more downregulated in glioma core region than that in the intermediate regions (**Figure 5D**). miR-143-5p was therefore selected for further experiments.

Next, a luciferase reporter assay was utilized to verify the putative bindings. According to the M&M section, two different types of OR7E156P or HIF1A 3’UTR luciferase reporter vectors were constructed, wild-type and mutant-type. These vectors were co-transfected in 293T cells with miR-143 mimics/inhibitor and the luciferase activity was determined. **Figure 5E** shows that the overexpression of miR-143 is dramatically inhibited, whereas the inhibition of miR-143 promoted wild-type luciferase reporter vectors’ luciferase activity; mutating the putative miR-143 binding site could eliminate the alterations within the luciferase activity. To further confirm the binding of miR-143 to OR7E156P and the 3’-UTR of HIF1A, AGO2 antibody was used to perform RIP assays. The anti-AGO2 immunoprecipitates containing miRNAs and their interacting RNA-components for the levels of miR-143 and OR7E156P were examined; as illustrated in **Figure 5F**, comparing with the anti-IgG immunoprecipitates, miR-143 and OR7E156P were more abundant in AGO2. Moreover, upon miR-143 overexpression, higher levels of OR7E156P were examined within the anti-AGO2 immunoprecipitates compared to the NC mimics group (**Figure 5F**); upon OR7E156P overexpression, lower levels of HIF1A mRNA were examined within the anti-AGO2 immunoprecipitates in comparison with the NC group (**Figure 5F**). In summary, miR-143 could potentially bind to OR7E156P and the 3’-UTR of HIF1A. Moreover, OR7E156P relieves the miR-143-induced inhibition upon H1F1A through competition with HIF1A for miR-143 binding.

### Dynamic Effects of OR7E156P and miR-143 on HUVEC Tube Formation and Glioma Cell Invasion Under Hypoxia

**Figure 6:**
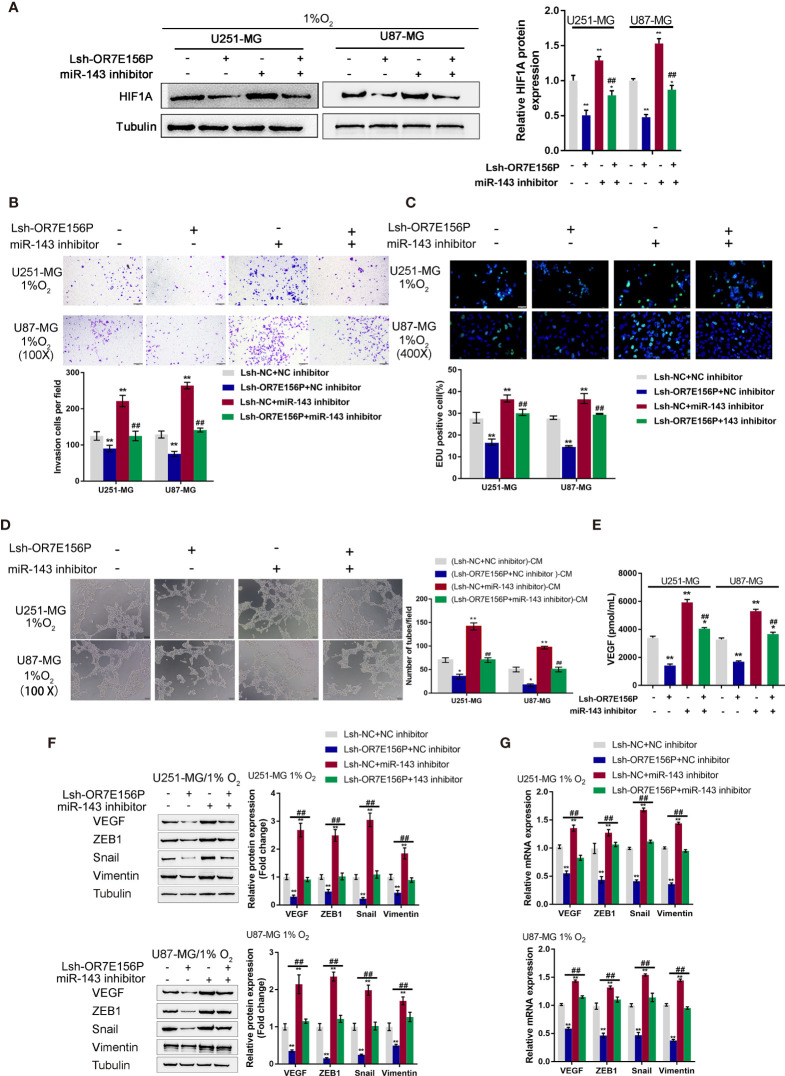
Dynamic effects of OR7E156P and miR-143 on HUVEC tube formation and glioma cell invasion **(A)** U251-MG and U87-MG cells were co-transfected with Lsh-OR7E156P and miR-143 inhibitor, exposed to 1% oxygen, and examined for the protein levels of HIF1A using Immunoblotting. **(B)** U251-MG and U87-MG cells were co-transfected with Lsh-OR7E156P and miR-143 inhibitor, exposed to 1% or 20% oxygen, and examined for the cell invasion by Transwell. **(C)** U251-MG and U87-MG cells were co-transfected with Lsh-OR7E156P and miR-143 inhibitor and exposed to 1% or 20% oxygen, and then examined for DNA synthesis ability by EdU assay; **(D)** U251-MG and U87-MG cells were co-transfected with Lsh-OR7E156P and miR-143 inhibitor and exposed to 1% or 20% oxygen. The culture medium was collected for HUVEC incubation. The tube formation ability of HUVEC was determined. **(E)** U251-MG and U87-MG cells were co-transfected with miR-143 inhibitor and Lsh-OR7E156P, exposed to 1% oxygen, and examined for the VEGF levels in culture medium by ELISA. *P < 0.05, **P < 0.01, compared to the control group; ^##^ *P* < 0.01, compared to the Lsh-NC + miR-143 inhibitor group. **(F)** U251-MG and U87-MG cells were co-transfected with Lsh-OR7E156P and miR-143 inhibitor, exposed to 1% oxygen, and examined for the protein levels of VEGF, ZEB1, Snail, and Vimentin by Immunoblotting. **(G)** U251-MG and U87-MG cells were co-transfected with Lsh-OR7E156P and miR-143 inhibitor, exposed to 1% oxygen, and examined for the mRNA levels of VEGF, ZEB1, Snail, and Vimentin by real-time qPCR. *P < 0.05, **P < 0.01, compared to the control group; ^##^ *P* < 0.01, compared to the Lsh-OR7E156P + NC inhibitor group.

After confirming the binding of miR-143 to OR7E156P and the 3’-UTR of HIF1A, respectively. The dynamic effects of the OR7E156P/miR-143 axis on HIF1A levels and glioma cells were subsequently investigated. Firstly, U251-MG and U87-MG cells were co-transfected with Lsh-OR7E156P and miR-143 inhibitor, exposed to 1% oxygen, and the protein levels of HIF1A were examined through Immunoblotting; as shown in **Figure 6A**, under hypoxia, hypoxia-induced surges in the protein contents of HIF-1α showed to be reduced by OR7E156P knockdown while they were further increased by miR-143 inhibition (**Figure 6A**); under hypoxia, miR-143 inhibition markedly attenuated the impacts of OR7E156P knockdown on HIF-1α protein levels (**Figure 6A**). Secondly, U251-MG and U87-MG cell lines were co-transfected with Lsh-OR7E156P and miR-143 inhibitor, exposed them to 1%, O_2_ and determined the dynamic effects of OR7E156P and miR-143 on glioma cell invasive capacity and DNA synthesis capacity. OR7E156P knockdown significantly downregulated, whereas miR-143 inhibition further upregulated hypoxia-induced cell invasion and DNA synthesis; miR-143 inhibition dramatically attenuated the effects of OR7E156P knockdown on hypoxia-induced cell invasion and DNA synthesis (**Figures 6B, C**). Thirdly, U251-MG and U87-MG cells were co-transfected with Lsh-OR7E156P and miR-143 inhibitor and exposed to 1% oxygen and the culture medium was collected for HUVEC incubation. The tube formation ability of HUVEC was subsequently determined. The tube formation ability of HUVECs in U251-MG (1%, Lsh-OR7E156P)-CM and U87-MG (1%, Lsh-OR7E156P)-CM was significantly inhibited while that in U251-MG (1%, miR-143 inhibitor)-CM and U87-MG (1%, miR-143 inhibitor)-CM was promoted; the inhibitory effects of OR7E156P knockdown on tube formation were significantly reversed by miR-143 inhibition (**Figure 6D**). Consistently, the knockdown of OR7E156P dramatically decreased, whereas the inhibition of miR-143 increased VEGF levels in the culture medium; the inhibition of miR-143 markedly attenuated the effects of OR7E156P knockdown on the culture medium’s VEGF levels (**Figure 6E**).

To investigate the underlying molecular mechanism, the protein levels and mRNA expression levels of VEGF, ZEB1, Snail, and Vimentin were examined using Immunoblotting and a real-time qPCR, respectively, under similar conditions. **Figure 6F** shows that hypoxia-induced VEGF, ZEB1, Snail, and Vimentin protein levels were further upregulated *via* miR-143 inhibition while partially downregulated *via* OR7E156P knockdown. Similarly, hypoxia-increased VEGF, ZEB1, and HIF1A mRNA expression levels were further upregulated *via* miR-143 inhibition while partially downregulated *via* OR7E156P knockdown (**Figure 6G**). miR-143 inhibition remarkably attenuated the effects of OR7E156P knockdown on these factors under conditions of hypoxia (**Figures 6F, G**). In summary, OR7E156P/miR-143 axis regulates HIF-1α downstream ZEB1, and VEGF signaling pathways, affecting glioma cell invasion.

### HIF1A Binds to the Promoter Region of miR-143 and Inhibit miR-143 Expression

**Figure 7:**
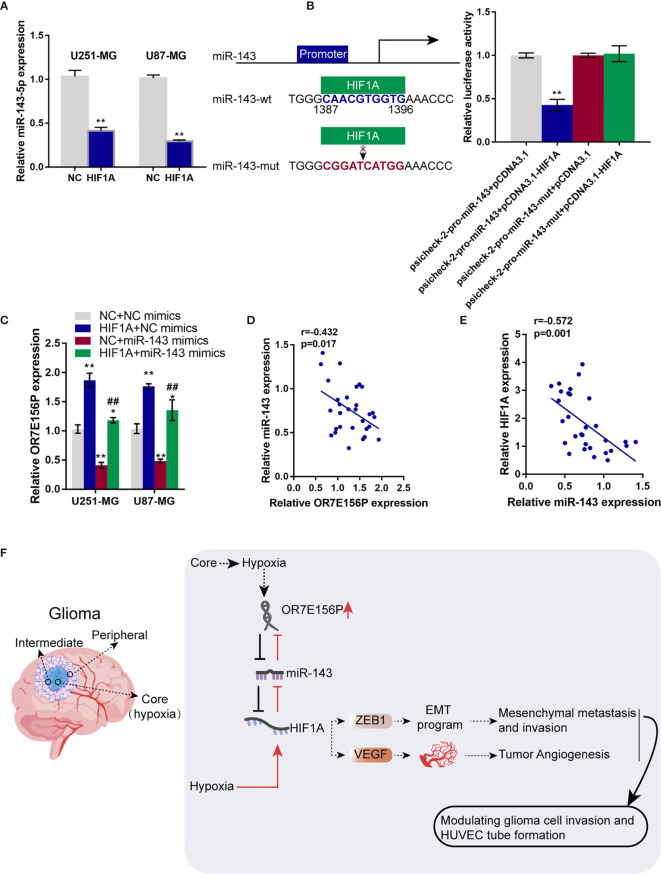
HIF1A binds to the promoter region of miR-143 and inhibit miR-143 expression **(A)** U251-MG and U87-MG cells were transfected with NC or HIF1A overexpression vector and examined for the expression of miR-143 by qRT-PCR. **(B)** Wild- and mutant-type miR-143 promoter luciferase reporter vectors, psicheck-2-pro-miR-143 and psicheck-2-pro-miR-143-mut, were constructed based on psiCheck-2 vectors. These vectors were co-transfected in 293T cells with pcDNA3.1/HIF1A and the luciferase activity was determined. **(C)** U251-MG and U87-MG cells were co-transduced with NC or HIF1A overexpression vector and NC mimics or miR-143 mimics and examined for the expression of OR7E156P by qRT-PCR. **(D, E)** The correlation of miR-143, OR7E156P, and HIF1A expression in tissue samples was analyzed using Pearson’s correlation analysis. **(F)** A schematic diagram showing the process of the OR7E156P/miR-143/HIF1A axis modulating glioma cell invasion through ZEB1 and HUVEC tube formation through VEGF. ^##^p < 0.01, *p < 0.05, **p < 0.01, compared to psicheck-2-pro-miR-143+pcDNA3.1 or NC+NC mimics group; ^##^p < 0.01, compared to NC+miR-143 mimics group.

Whether HIF1A could affect miR-143 expression in return was ultimately examined. U251-MG and U87-MG cells were transduced with NC or HIF1A overexpression vector and examined for the expression of miR-143 by qRT-PCR. As illustrated in **Figure 7A**, HIF1A overexpression significantly downregulated miR-143 expression. Since the online tool predicted a possible HIF1A binding site in miR-143 promoter region, wild- and mutant-type miR-143 promoter luciferase reporter vectors were subsequently constructed, psicheck-2-pro-miR-143 and psicheck-2-pro-miR-143-mut, based on psiCheck-2 vectors. These vectors were co-transfected in 293T cells with pcDNA3.1/HIF1A and the luciferase activity was determined. As shown in **Figure 7B**, when co-transfected with psicheck-2-pro-miR-143, HIF1A overexpression significantly suppressed the luciferase activity; when co-transfected with psicheck-2-pro-miR-143-mut, HIF1A overexpression failed to alter the luciferase activity. U251-MG and U87-MG cells were co-transduced with NC or HIF1A overexpression vector and NC mimics or miR-143 mimics and examined for the expression of OR7E156P by qRT-PCR. HIF1A overexpression upregulated, whereas miR-143 overexpression downregulated OR7E156P expression; the promotive effects of HIF1A overexpression on OR7E156P were partially reversed by miR-143 overexpression (**Figure 7C**). In tissue samples, miR-143 was negatively correlated with OR7E156P and HIF1A, respectively (**Figures 7D, E**).

## Discussion

An abnormally upregulated lncRNA, OR7E156P, in glioma was conclusively identified. Silencing of OR7E156P inhibited cell invasion and DNA synthesis *in vitro* and tumor growth *in vivo*. OR7E156P was intricately linked with the HIF1A pathway. Hypoxia could induce OR7E156P expression, whereas OR7E156P silencing decreased HIF1A protein levels under hypoxic conditions. Hypoxia promoted glioma cell invasion and DNA synthesis, and HUVEC tube formation, whereas OR7E156P silencing partially reversed the cellular effects of hypoxia. miR-143 directly targeted OR7E156P and HIF1A, respectively. miR-143 inhibition increased HIF1A protein levels, promoted glioma cell invasion and DNA synthesis, and enhanced HUVEC tube formation, whereas OR7E156P silencing partially reversed the cellular effects of miR-143 inhibition. HIF1A targeted the promoter region of miR-143 and inhibited miR-143 expression.

During glioma initiation and development, numerous genes, including lncRNAs, could be altered, which were suspected of participating in the chemoresistance of glioma. For example, by network analysis and survival analysis in cerebral lower grade glioma, Xiong et al. (36) demonstrated the risk score model based on the 10 genes that could moderately predict glioma patients’ overall survival. In this study, differentially expressed lncRNAs in glioma and non-cancerous tissue samples were compared by analyzing online datasets, and found that lncRNA OR7E156P was significantly upregulated in glioma tissue samples. Moreover, the upregulated OR7E156P was verified in glioma cell lines and glioma tissues. The abnormal upregulation of OR7E156P suggests its potential role in glioma pathogenesis and the biological function of OR7E156P in glioma remains unclear as of yet. As speculated, the silencing of OR7E156P inhibited glioma cell invasion and DNA synthesis *in vitro*, and inhibited tumor growth in nude mice *in vivo*.

Hypoxia is a hallmark characteristic of glioblastoma development. In gliomas, more immature cells are located in the inner core and in the intermediate layer of the tumor mass, and more committed cells are distributed in the peripheral and neovascularization area (37). Due to the hyperproliferation of glioma cells, the oxygen consumption outstrips the blood supply, resulting in intratumoral necrosis and hypoxic signaling induction (38). Extensive hypoxic regions in glioblastomas contribute to the highly malignant phenotype of these tumors. Hypoxic regions of glioblastoma exacerbate the prognosis and clinical outcomes of the patients as hypoxic tumor cells are resistant to chemo- and radiation therapy and are also protected by the hypoxia-induced malfunctional vasculature (39–41). Under hypoxic conditions, the expression levels of OR7E156P in glioma cells were significantly upregulated, further suggesting the role of OR7E156P in the correlation with a hypoxic microenvironment. Moreover, an online signaling pathway enrichment analysis suggested the possible association of OR7E156P with the HIF1A signaling pathway.

HIF-1α is a central mediator of hypoxia-related signaling, was increased within a number of malignancies mainly through hypoxia-induced protein stabilization (42). Within gliomas, HIF-1α primarily is localized in pseudopalisading cells around areas of necrosis and in tumor cells infiltrating the brain at the tumor margin (24). Inside the anoxic and in the intermediate area of the glioma tumor mass, the highest level of HIF-1α was observed (20). Herein, in glioma cells, hypoxia stimulation markedly increased HIF1A expression. Hypoxia-induced HIF1A upregulation in glioma cells was significantly reversed by silencing OR7E156P, suggesting that OR7E156P might affect HIF1A, subsequently modulating glioma cell response to hypoxia. As speculated, hypoxia promoted cancer cell invasion and DNA synthesis, whereas OR7E156P silencing significantly attenuated the promotive effects of hypoxia on cancer cells. In nude mice *in vivo*, HIF1A overexpression reversed the inhibitory effects of OR7E156P knockdown on tumor growth. Consistent with our observations, Huang et al. identified hypoxia as a factor which enhanced the migration and invasion of U87 cells through the PI3K/Akt/mTOR/HIF1A pathway (43). Rodriguez et al. demonstrated that hypoxia-induced USP22-BMI1 axis promoted the stemness and malignancy of glioma stem cells through the regulation of HIF1A (44). The above outcomes revealed that hypoxia influences the progression of glioma by regulating HIF1A.

To further investigate the underlying mechanism, the study continued to screen for miRNAs that potentially mediate the cross-talk between OR7E156P. HIF1A and miR-143 was subsequently selected. As previously reported, miR-143 could exert a tumor-suppressive effect. Within colorectal carcinoma, miR-143 targets insulin-like growth factor-I receptor to inhibit tumor growth and vascularization and to re-sensitize the tumor to oxaliplatin (45). Within cervical carcinoma, miR-143 targets H1F1A to inhibit cancer cell proliferation and apoptosis (46). Herein, miR-143 could bind to both OR7E156P and HIF1A. By competing with HIF1A for miR-143 binding, OR7E156P counteracted miR-143-mediated suppression on HIF1A. Consistently, miR-143 knockdown within glioma cells dramatically reversed the roles of OR7E156P knockdown in HIF1A, ZEB1, and VEGF expression. Regarding the cellular functions, miR-143 knockdown attenuated the roles of OR7E156P knockdown in glioma cell invasion, proliferation, and HUVEC tube formation under hypoxia. Interestingly, by both online tool prediction and experimental examination, HIF1A could bind to the promoter region of miR-143, inhibiting miR-143 expression. When HIF1A was overexpressed, miR-143-mediated suppression on OR7E156P expression was attenuated.

Altogether, a regulatory axis consisting of OR7E156P, miR-143, and HIF1A, which is deregulated in glioma was identified, and the process of the OR7E156P/miR-143/HIF1A axis modulating glioma cell invasion through ZEB1 and HUVEC tube formation through VEGF was ascertained (**Figure 7F**).

## Data Availability Statement

The original contributions presented in the study are included in the article/**Supplementary Material**. Further inquiries can be directed to the corresponding author.

## Ethics Statement

This study was performed according to the recommendations of the Declaration of Helsinki and was approved by the ethics committee of XiangYa Hospital, approval No.201703741(human). The patients/participants provided their written informed consent to participate in this study. This study was performed according to the recommendations of the Declaration of Helsinki and was approved by the ethics committee of XiangYa Hospital, approval No.201703740 (animal).

## Author Contributions

HZ and PD performed the mainly investigation and wrote the manuscript. RP performed the bioinformatic analysis. GP, JY, and DL performed the molecular experiments. YL and XM collected the samples and assisted with the experiments. YWL administrated the whole project and editing the manuscript. All authors contributed to the article and approved the submitted version.

## Funding

This study was supported by National Natural Science Foundation of China, (Grant No: 81702490) and Natural Science Foundation of Xinjiang Uygur Autonomous Region (2017D01C247).

## Conflict of Interest

The authors declare that the research was conducted in the absence of any commercial or financial relationships that could be construed as a potential conflict of interest.

## Publisher’s Note

All claims expressed in this article are solely those of the authors and do not necessarily represent those of their affiliated organizations, or those of the publisher, the editors and the reviewers. Any product that may be evaluated in this article, or claim that may be made by its manufacturer, is not guaranteed or endorsed by the publisher.

## Supplementary Material

Supplementary Figure 1 Differentially expressed genes in glioma and non-cancerous tissues based on TCGA database.

Supplementary Figure 2 Differentially expressed genes in glioma and non-cancerous tissues based on GSE42658.

Supplementary Figure 3 The effects of OR7E156P on the primary human glioma cells’ proliferation and invasion. **(A)** Phenotypes of the primary human glioma cells captured by microphotography are displayed. **(B)** Immunocytochemistry for GFAP and S100B was done in primary human glioma cells (glioma-PC), U251-MG and U87-MG cells. **(C)** OR7E156P silencing was achieved in primary human glioma cells by infecting cells with lentivirus containing short hairpin RNA for OR7E156P (Lsh1-OR7E156P or Lsh2-OR7E156P). Lsh-NC was infected as a negative control. The silencing efficiency was verified by qRT-PCR. Then, primary human glioma cells were infected with Lsh1-OR7E156P or Lsh-NC and examined for cell invasion by Transwell assay **(E)**; DNA synthesis capacity by EdU assay **(F)**.

Supplementary Figure 4 The BioCarta tool from (https://www.gsea-msigdb.org/gsea/) was used to verify the association between OR7E156P and HIF signaling pathway.
