# Supplementary figures and images for: Long Noncoding RNA OR7E156P/miR-143/HIF1A Axis Modulates the Malignant Behaviors of Glioma Cell and Tumor Growth in Mice
Source: Front Oncol. 2021 Aug 6;11:690213. doi: 10.3389/fonc.2021.690213 (PMC8377393; doi:10.3389/fonc.2021.690213)

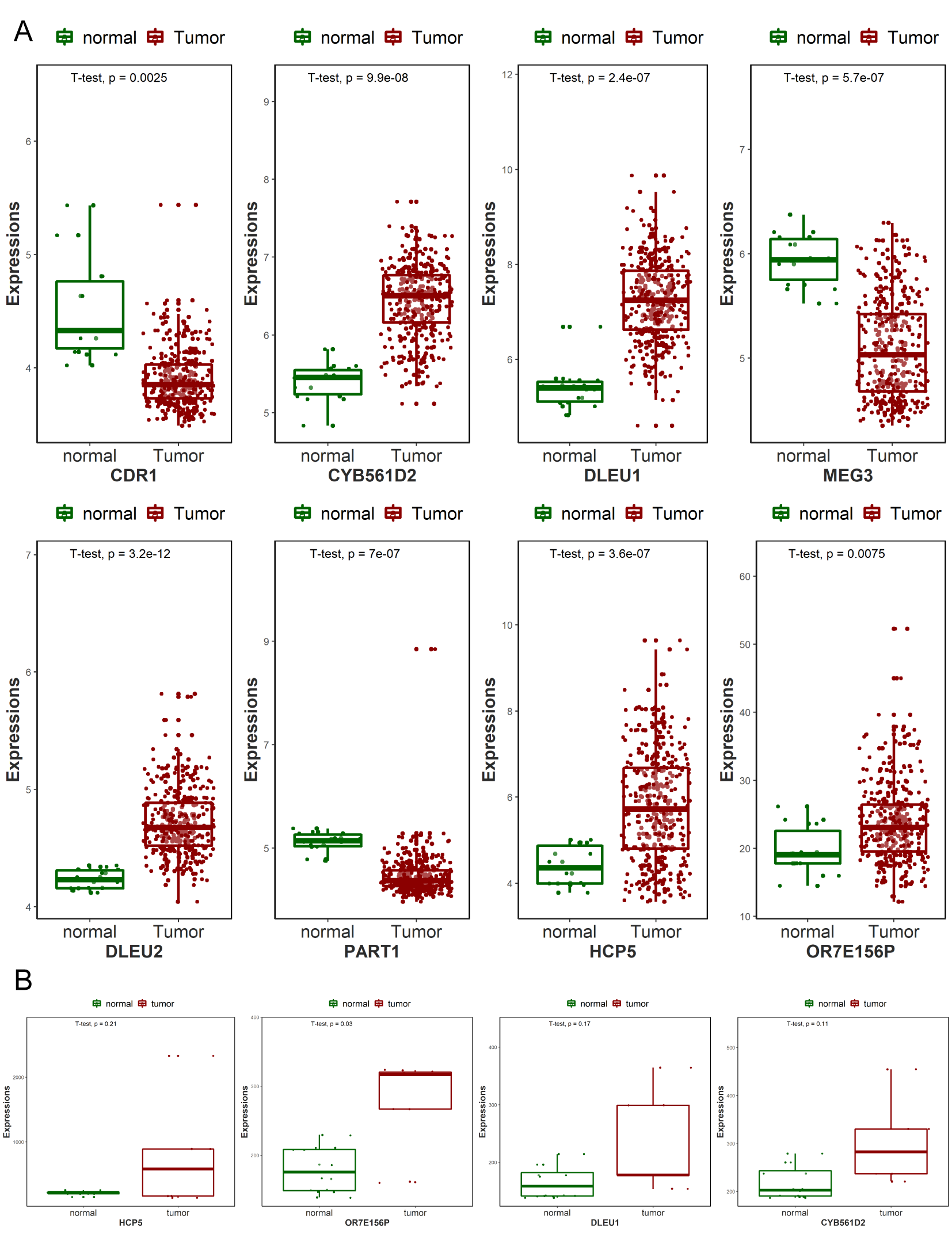

Supplement: Supplementary Figure 1 — Differentially expressed genes in glioma and non-cancerous tissues based on TCGA database. [file Image_1.tif]

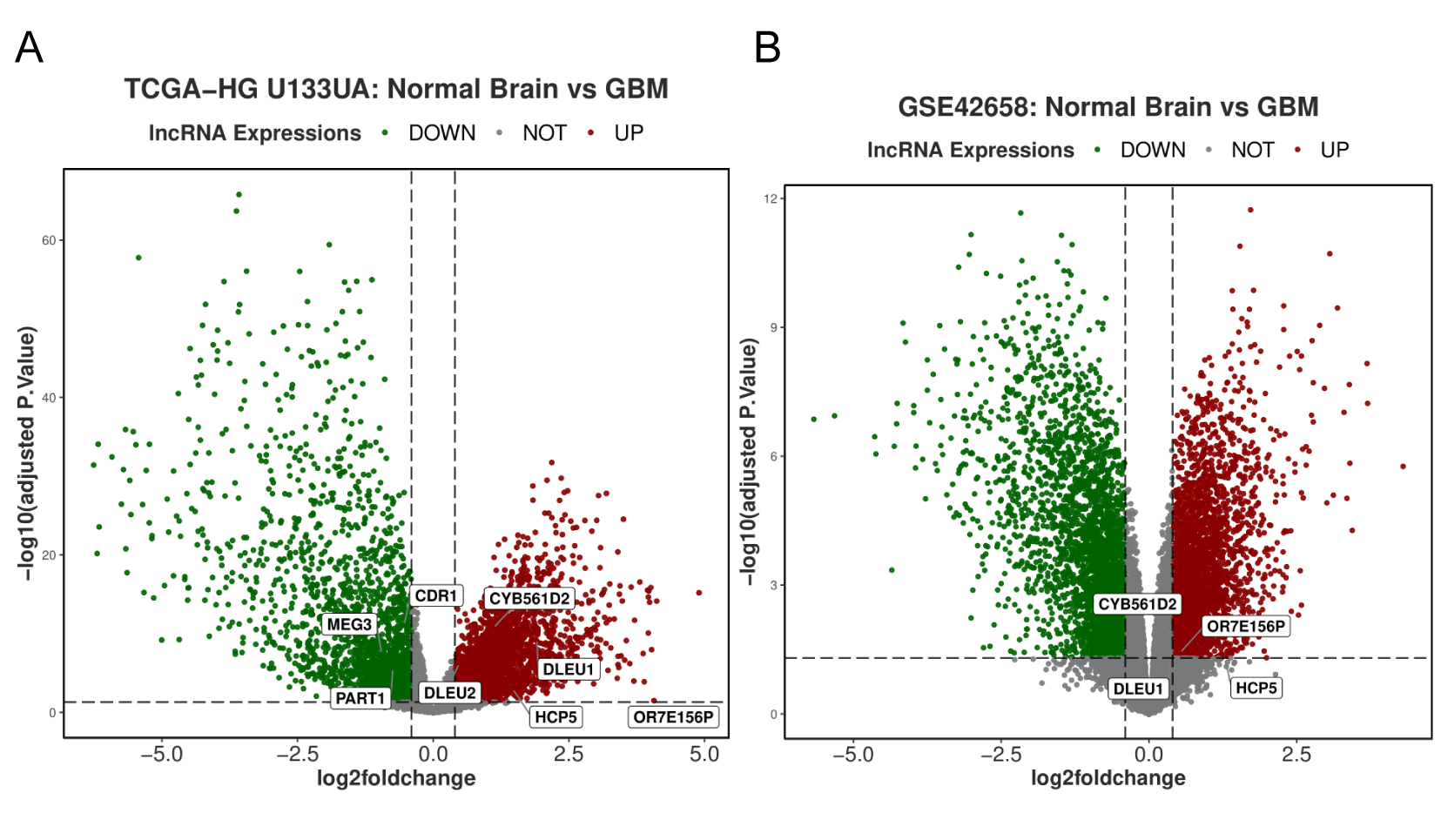

Supplement: Supplementary Figure 2 — Differentially expressed genes in glioma and non-cancerous tissues based on GSE42658. [file Image_2.tif]

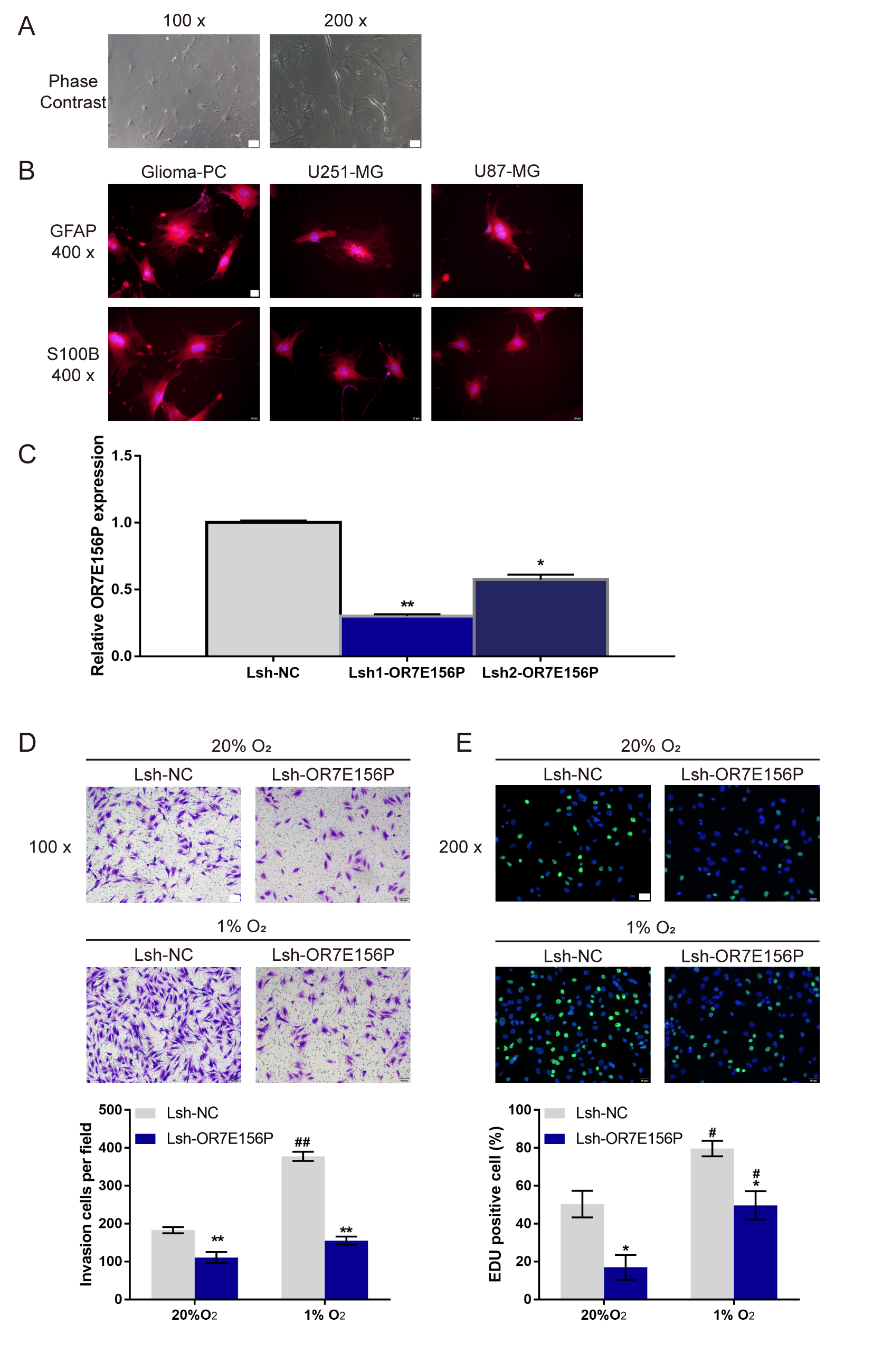

Supplement: Supplementary Figure 3 — The effects of OR7E156P on the primary human glioma cells’ proliferation and invasion. (A) Phenotypes of the primary human glioma cells captured by microphotography are displayed. (B) Immunocytochemistry for GFAP and S100B was done in primary human glioma cells (glioma-PC), U251-MG and U87-MG cells. (C) OR7E156P silencing was achieved in primary human glioma cells by infecting cells with lentivirus containing short hairpin RNA for OR7E156P (Lsh1-OR7E156P or Lsh2-OR7E156P). Lsh-NC was infected as a negative control. The silencing efficiency was verified by qRT-PCR. Then, primary human glioma cells were infected with Lsh1-OR7E156P or Lsh-NC and examined for cell invasion by Transwell assay (E); DNA synthesis capacity by EdU assay (F). [file Image_3.tif]

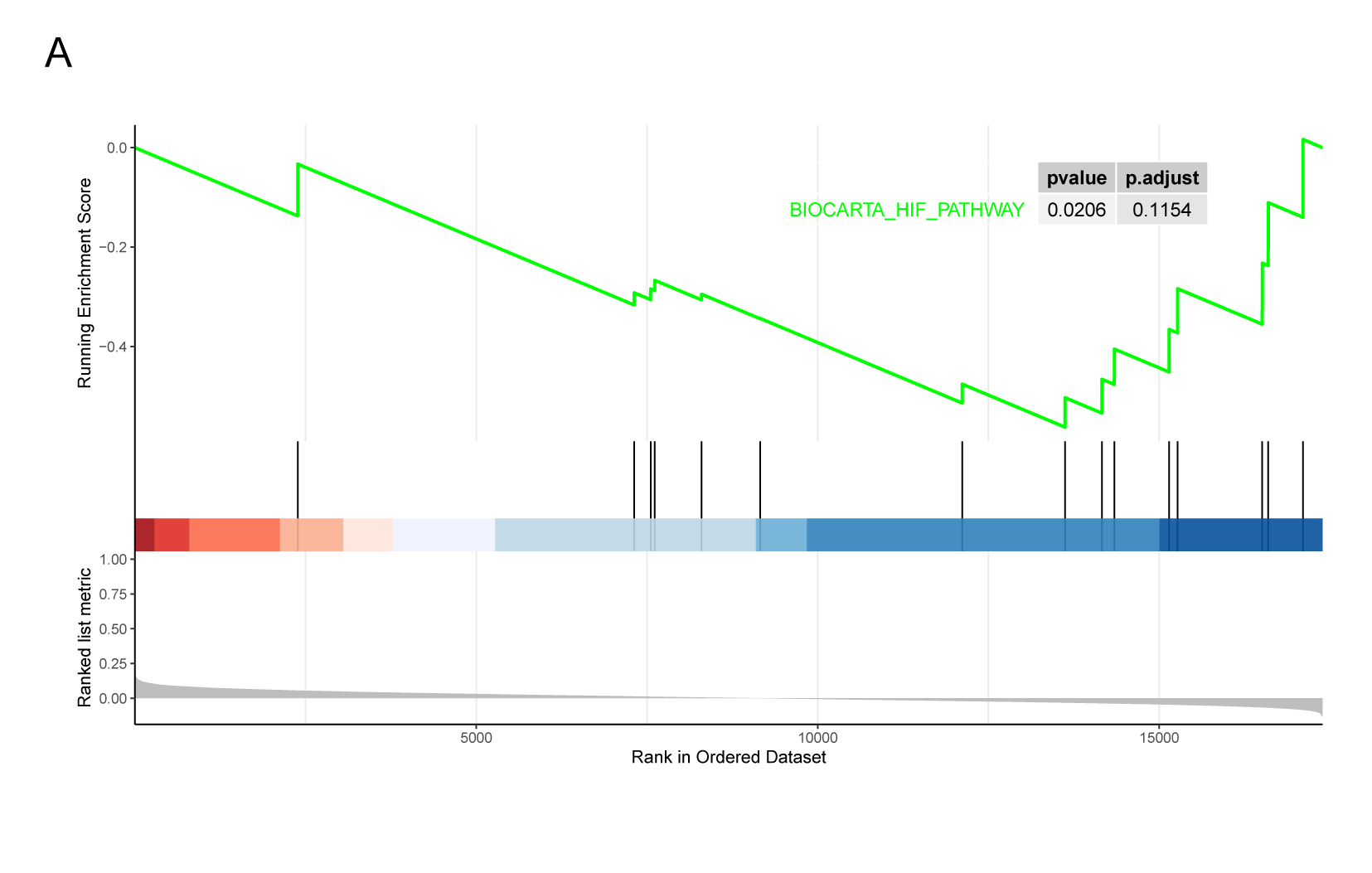

Supplement: Supplementary Figure 4 — The BioCarta tool from (https://www.gsea-msigdb.org/gsea/) was used to verify the association between OR7E156P and HIF signaling pathway. [file Image_4.tif]
